# Health-related quality of life of young adults with Turner syndrome following a long-term randomized controlled trial of recombinant human growth hormone

**DOI:** 10.1186/1471-2431-11-49

**Published:** 2011-05-29

**Authors:** Shayne P Taback, Guy Van Vliet

**Affiliations:** 1 Departments of Pediatrics and Child Health and Community Health Sciences, University of Manitoba, Winnipeg, MB, R3T 2N2, Canada; 2 Endocrinology Service and Research Center, Sainte-Justine Hospital and Department of Pediatrics, University of Montreal, Montreal, QC, H3T 1C5, Canada; 3 Canadian Pediatric Endocrinology Group, Canada

## Abstract

**Background:**

There are limited long-term randomized controlled trials of growth hormone (GH) supplementation to adult height and few published reports of the health-related quality of life (HRQOL) following treatment. The present follow-up study of young adults from a long-term controlled trial of GH treatment in patients with Turner syndrome (TS) yielded data to examine whether GH supplementation resulted in a higher HRQOL (either due to taller stature or from the knowledge that active treatment and not placebo had been received) or alternatively a lower HRQOL (due to medicalization from years of injections).

**Methods:**

The original trial randomized 154 Canadian girls with TS aged 7-13 years from 13 centres to receive either long-term GH injections at the pharmacologic dose of 0.3 mg/kg/week or to receive no injections; estrogen prescription for induction of puberty was standardized. Patients were eligible for the follow-up study if they were at least 16 years old at the time of follow-up. The instrument used to study HRQOL was the SF-36, summarized into physical and mental component scales (PCS and MCS); higher scores indicate better HRQOL.

**Results:**

Thirty-four of the 48 eligible participants (71%) consented to participate; data were missing for one patient. Both groups (GH and no treatment) had normal HRQOL at this post-treatment assessment. The GH group had a (mean ± SD) PCS score of 56 ± 5; the untreated group 58 ± 4; mean score for 16-24 year old females in the general population 53.5 ± 6.9. The GH group had a mean MCS score of 52 ± 6; the untreated group 49 ± 13; mean score for 16-24 year old females in the general population 49.6 ± 9.8. Secondary analyses showed no relationship between HRQOL and height.

**Conclusions:**

We found no benefit or adverse effect on HRQOL either from receiving or not receiving growth hormone injections in a long-term randomized controlled trial, confirming larger observational studies. We suggest that it remains ethically acceptable as well as necessary to maintain a long-term untreated control group to estimate the effects of pharmacological agents to manipulate adult height. Young adult women with TS have normal HRQOL suggesting that they adjust well to their challenges in life.

**Trial Registration:**

ClinicalTrials.gov Identifier NCT00191113.

## Background

Growth hormone supplementation of short children without growth hormone deficiency is increasing worldwide. However, long-term randomized trials that remain controlled until adult height has been reached are rare, as are published follow-up data on the quality of life of the participants [1]. We previously identified several barriers to such long-term trials including physician discomfort with random assignment of children to an untreated long-term control group [1]. However, two long-term randomized controlled trials of growth hormone supplementation [2,3] have been conducted in girls with Turner syndrome [4], a population in whom growth hormone supplementation is widely used [5].

A recent literature search (details not shown) on the health-related quality of life in young adults with Turner syndrome who received growth hormone revealed no published randomized controlled trials but yielded two observational studies that used the SF-36, a widely used, short, generic, psychometrically sound measure of subjective health status [6]. Carel et al surveyed 568 adult women from a mandatory population-based treatment registry, mean age 22.6 years, mean height 150.9 cm, 69% response rate [7]. There were no effects of height or estimated height gain from treatment on health-related quality of life. Bannink et al surveyed 49 adult women who had been treated with growth hormone in previous treatment studies, mean age 19.6 years; mean height 160.7 cm [8]. The group had normal health-related quality of life, although estimated height gain was positively correlated to one subscale: "role limitations due to physical health problems". Earlier, Busschbach et al surveyed twenty-five short adult women with Turner syndrome not treated with growth hormone who were the first to respond to their request to participate [9]. They found normal scores on the six dimensions of the Nottingham Health Profile, an earlier measure of health-related quality of life. They also found that 68% would like to be taller and 44% were willing to trade-off a small proportion of their total lifespan to that end. Of the latter sub-group, the mean time trade-off in exchange for normalization of height to the general population average was 4.2% of lifespan, smaller than the total group's trade-off for not having infertility, 9% of lifespan. Boman et al also used the Nottingham Health Profile in relatively older women with Turner syndrome, mean age 37 years [10]. They reported significantly lower scores in only one dimension, social isolation. Effects of height or treatment with growth hormone were not reported. Finally, Naess et al surveyed 80 relatively older women with Turner syndrome, mean age 34 years; mean height 152.8 cm, 50% response rate from one specialty clinic and one voluntary registry [11]. They reported significantly lower scores in two SF-36 subscales: "physical functioning" and "general health". Associations of height or treatment with growth hormone with SF-36 scores were not reported. They suggested these significant findings may be related to the older age at testing compared with the other studies using the SF-36.

We performed a follow-up study of one of the two randomized controlled trials [2] and included the collection of health-related quality of life data on these young women. The unblinded design of the trial allowed us to not only test health-related quality of life hypotheses about treatment outcomes but also treatment group assignment effects. Specifically, we examined whether growth hormone supplementation resulted in a higher health-related quality of life (either due to taller stature or from the knowledge that active treatment and not placebo had been received) or alternatively a lower health-related quality of life (due to medicalization owing to the years of injections).

## Methods

The original trial (ClinicalTrials.gov identifier NCT00191113) randomized 154 Canadian girls with Turner syndrome aged 7-13 years from 13 centers, using centralized randomization, to receive either long-term growth hormone injections at the pharmacologic dose of 0.3 mg/kg/week divided into six injections per week or to receive no injections [2]. The participants were therefore not blinded to treatment assignment but were otherwise treated similarly. Owing to the high frequency of ovarian failure in Turner syndrome, their treatment included a standardized prescription for induction of puberty with daily low dose estrogen (0.0025 mg ethinyl estradiol) after reaching both a chronologic age of 13 years and a minimum treatment with growth hormone of one year if in the treated group. After one year of initial estrogen treatment, this dose was increased to 0.005 mg for one additional year before cyclic estrogen and progesterone replacement was instituted. The primary results of the original trial were that growth hormone supplementation increased adult height in women with Turner Syndrome by 7.2 cm on average (95% CI 6.0 - 8.4 cm) with high individual variability in the magnitude of response.

The present study was conducted at six of the original thirteen centers from October 1999--February 2001. The predominant reason for non-participation of seven centers was lack of clinician-investigator time or resources to dedicate to a multicenter project at that time. Participants were eligible if they were at least 16 years of age within the study window and had participated in the original study at one of the six participating centers. The present study received institutional research ethics board approval initially from the University of Manitoba Research Ethics Board and subsequently from the ethics committee at each of the other participating sites. Written informed consent was obtained for each participant.

The SF-36 was used to measure health-related quality of life [6]. Its 36 questions cover eight different health concepts: physical functioning, role limitations due to physical problems, bodily pain, general health, vitality, social functioning, role limitations due to emotional problems, and mental health. These eight concepts can be validly summarized into two component scales: physical (PCS) and mental (MCS) as long as findings specific to one of the eight health concepts are not obscured [12]. The PCS and MCS are expressed as norm-based scores with a population mean of 50, and standard deviation of 10; higher scores indicating better health. The instrument was administered, in English or French, as a questionnaire for self-completion prior to the remainder of the detailed questionnaire on participant demographics, medical history, risk factors for osteoporosis and auxologic and bone densitometry measurements (to be published separately). The SF-36 data were scored electronically by the QualityMetric scoring service (QualityMetric Inc., RI).

Continuous variables were summarized by mean and 95% confidence interval (CI) and categorical variables by frequencies and 95% CIs. Bivariate comparisons were also computed. The SAS (SAS Institute, NC) multiple linear regression procedure, PROC REG, was used to regress the SF-36 outcomes data on treatment assignment, adult height, body mass index, and spontaneous menarche to compute regression coefficients and their 95% CI. In addition to the intent-to-treat analysis, a planned sensitivity analysis was run using the participants' "as-treated" status; post-hoc exploratory analyses were also done.

## Results

Thirty-four of the 48 eligible participants (71%) consented to participate in the present follow-up study; however data were missing for one patient. The 21 participants randomized to growth hormone were 20.0 years of age (SD 2.4), 148.9 cm tall (SD 5.7) and had a body mass index of 25.7 kg/m2 (SD 4.9); 19 were adherent to growth hormone treatment long-term. The 12 participants randomized to no injections were 20.2 years of age (SD 2.1), 143.7 cm tall (SD 6.1) and had a body mass index of 24.2 (SD 3.7); 10 of the 12 had never received growth hormone injections. Both study groups had a normal health-related quality of life similar to the norms for females aged 18-24 years from the general Canadian population [13]; treatment group: PCS 56 (SD 4.9), MCS 52 (SD 6.3) and control group: PCS 58 (SD 4.2), MCS 49 (SD 13.2) (see Table 1). The individual subscales were also normal for both groups.

**Table 1:** Participant characteristics and HR-QOL results

	Randomized to growth hormone injections until adult height	Randomized to no injections
Number	21	12
Age (years)	20.0 (SD 2.4)	20.2 (SD 2.1)
Height (cm)	148.9 (SD 5.7)	143.7 (SD 6.1)
BMI (kg/m^2^)	25.7 (SD 4.9)	24.2 (SD 3.7)
Adherence	20 received injections	10 did not receive growth hormone injections
SF-36 PCS	56 (SD 4.9)	58 (SD 4.2)
SF-36 MCS	52 (SD 6.3)	49 (SD 13.2)
SF-36 subscales with norms		
Physical Functioning	54 (SD 5.9)	55 (SD 2.7)
Role Physical	56 (SD not calculable)	55 (SD 2.8)
Bodily Pain	58 (SD 5.4)	59 (SD 4.7)
General Health	53 (SD 6.4)	56 (SD 6.9)
Vitality	56 (SD 7.3)	55 (SD 7.8)
Social Functioning	53 (SD 7.5)	51 (SD 9.9)
Role Emotional	54 (SD 5.0)	49 (SD 11)
Mental Health	52 (SD 5.7)	50 (SD 11)

Separating the 33 patients by adult height revealed no association with SF-36 score. The 13 patients who were taller than the average (mean height 153.6 cm, SD 3.9) had a PCS of 56.6 (SD 2.6), and a MCS of 52.3 (SD 5.1), while the 20 patients shorter than the average (mean height 142.8 cm, SD 2.9) had a PCS of 56.9 (SD 5.7), and a MCS of 50.1 (SD 11.4). Secondary multivariate analyses also showed no statistically significant variables when regressing either the PCS or MCS on age, body mass index, presence of spontaneous menarche, or growth hormone trial treatment group. For the latter variable, growth hormone trial treatment group, the mean effect on MCS was 4.6 points: 95% CI -2.7--12 and on PCS, -2.3 points; 95% CI -5.9--1.3.

## Discussion

Health-related quality of life is an increasingly popular outcome as it subjectively measures how individuals feel rather than what the objective outcomes imply they ought to feel [6]. The similarity in scores between the groups suggests that, for girls with Turner syndrome, the impact of short stature and its treatment on health-related quality of life as young adults may have been overemphasized. It should be noted that the SF-36 is a generic health-related quality of life instrument that therefore may be unable to detect specific effects on quality of life due to height differences that do not affect the global physical or mental scores. Although our study sample was small, the groups we studied were formed by randomization and support the lack of a relationship of adult height to PCS or MCS seen in a larger observational study [7]. However, a sample size of 64 experimental participants would have been required to exclude a five point change on PCS or MCS under standard assumptions: two-sided test, alpha = 0.05, 80% power [12]. Calculating the effect size and 95% confidence interval of growth hormone trial group using an analysis of variance revealed that we have excluded a meaningful clinical effect of growth hormone treatment on PCS but not MCS. We do have limited data on the similarity of participants to non-participants; specifically the adult height data for our participants by treatment group, 148.9 cm versus 143.7 cm, are very similar to the adult height results one year after protocol completion for the parent trial, 149.0 cm versus 142.2 cm [2].

## Conclusions

We found no benefit or adverse effect on health-related quality of life in young adult women with Turner syndrome either from receiving or not receiving growth hormone injections in a long-term randomized controlled trial. However, we lacked the power to definitively exclude a clinically relevant benefit of growth hormone on MCS or a clinically relevant harm on PCS. We suggest that it remains ethically acceptable as well as necessary to maintain a long-term untreated control group to estimate the effects of pharmacological agents to manipulate adult height. This would apply equally to new agents and to new adult height indications for existing agents.

## Competing interests

Both authors have served as investigators for multicentre trials and observational studies of growth hormone products funded by pharmaceutical manufacturers. Currently, SPT is a local principal investigator (University of Manitoba) for Genesis, an observational study of growth hormone treatment operated and funded by Eli Lilly. Currently, GVV is a local co-investigator for Genesis (Université de Montréal).

## Authors' contributions

SPT participated in the conception, design, analysis, interpretation, and drafting of the manuscript. GVV participated in the conception, design, analysis, interpretation, and revision of the manuscript. Both authors read and approved the final manuscript.

## Pre-publication history

The pre-publication history for this paper can be accessed here:

http://www.biomedcentral.com/1471-2431/11/49/prepub
